# Supplementary material for: Single cell transcriptomes and multiscale networks from persons with and without Alzheimer’s disease
Source: Nat Commun. 2024 Jul 10;15:5815. doi: 10.1038/s41467-024-49790-0 (PMC11237088; doi:10.1038/s41467-024-49790-0)
Supplement: Supplementary file 2 — Supplementary Information [file 41467_2024_49790_MOESM2_ESM.pdf]

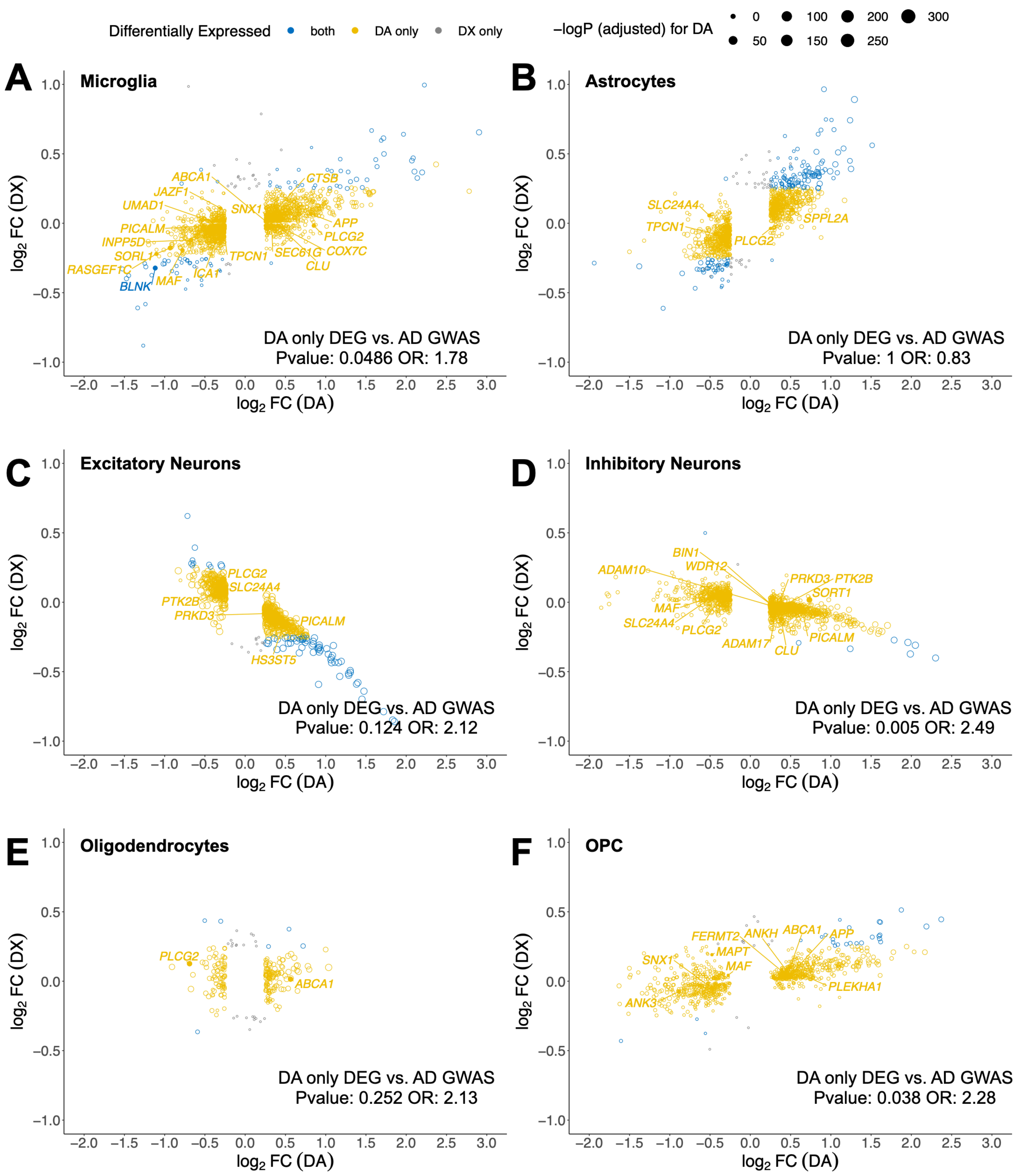

Supplementary Figure 1: Log2 fold change for differentially expressed genes (DEGs) obtained by two different comparisons obtained for major cell populations. DA: Differentially abundant vs non differentially abundant nuclei clusters; DX: AD nuclei vs age-matched control nuclei clusters. Circle size is proportional to the -log of the adjusted pvalue observed in the DA contrast. AD-associated[25] signature genes are labelled. P-value and Odds Ratio (OR) are shown for overlap between genes identified as DEG only using the DA approach (orange) and a set of AD GWAS associated candidate genes[25] (two-sided Fisher's Exact Test). OPC: Oligodendrocyte Precursor Cells.

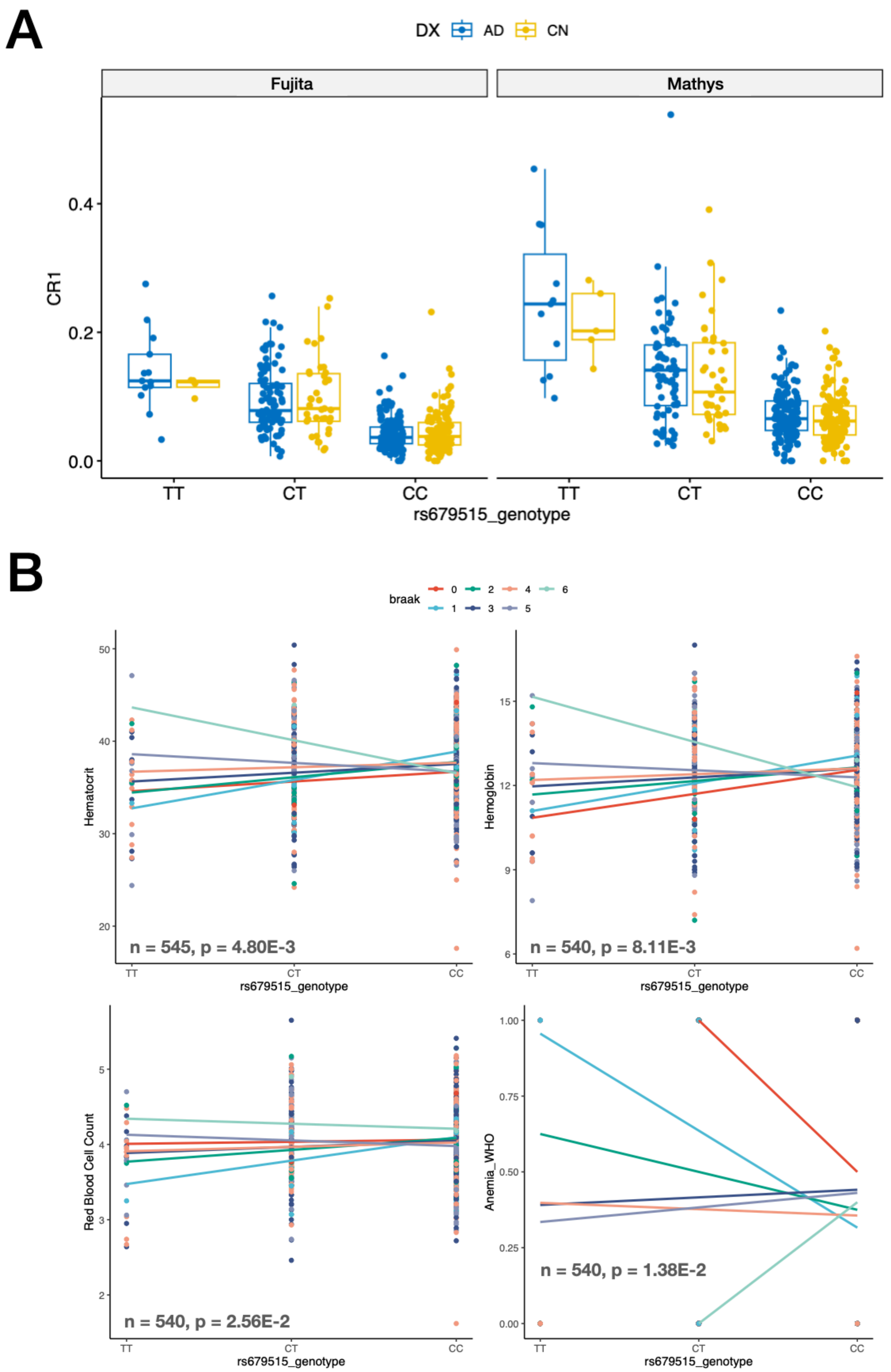

Supplementary Figure 2: Relationship between rs679515 genotype and *CR1* gene expression in oligodendrocytes and antemoterm hematological measurements in the ROSMAP cohorts. **(A)** CR1 expression stratified by AD diagnosis (Diagnosis was dichotomized by NIA-Reagan score). **(B)** Hematological parameter associations with rs679515 genotype, stratified by Braak score. Pvalues for statistical interaction between rs679515 dosage and Braak score shown. Sample number (n) shown. Detailed statistical metrics for each model are reported in Supplementary Table 5.

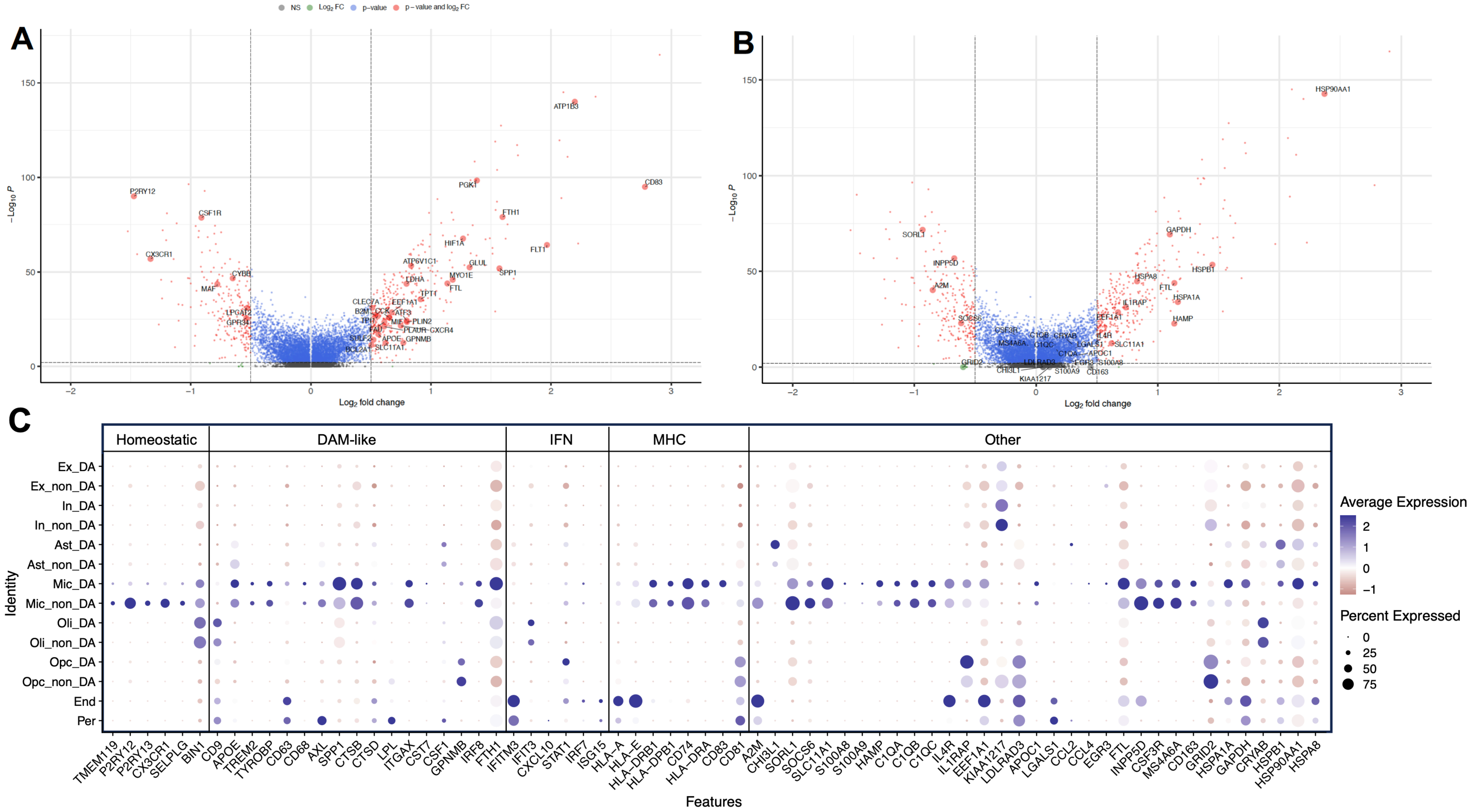

Supplementary Figure 3: DEGs identified in the DA9 population within microglia. DAM gene signatures are labelled. Only those with absolute $log_2FC > 0.5$ and adjusted $P < 0.05$ are colored. **(A)** human DAM gene signatures; **(B-C)** other human microglial gene signatures reported in literature[38].

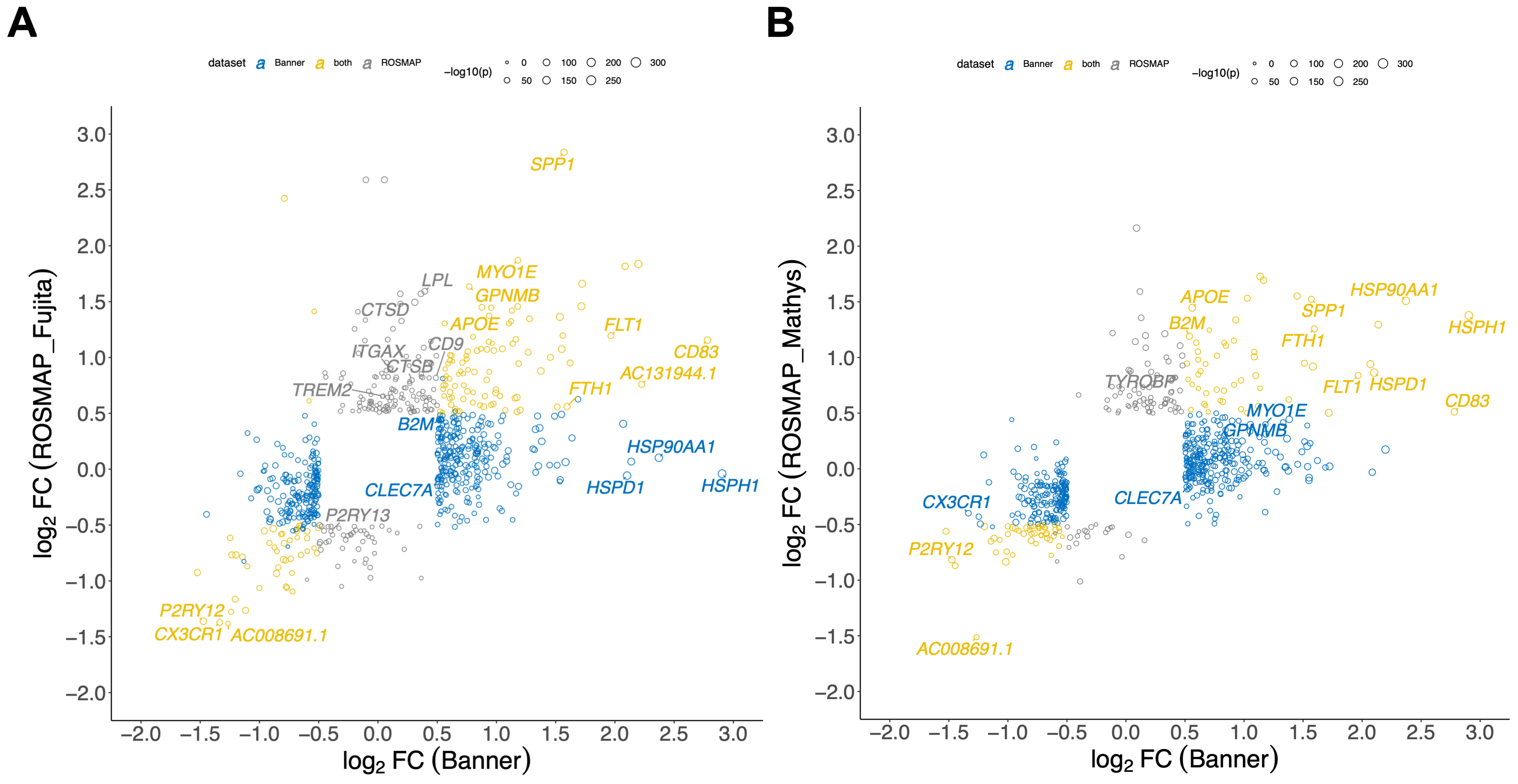

Supplementary Figure 4: Log2 fold change for DEGs obtained by DA vs non-DA cells in microglia in two datasets (Banner vs ROSMAP) analysed in this study.

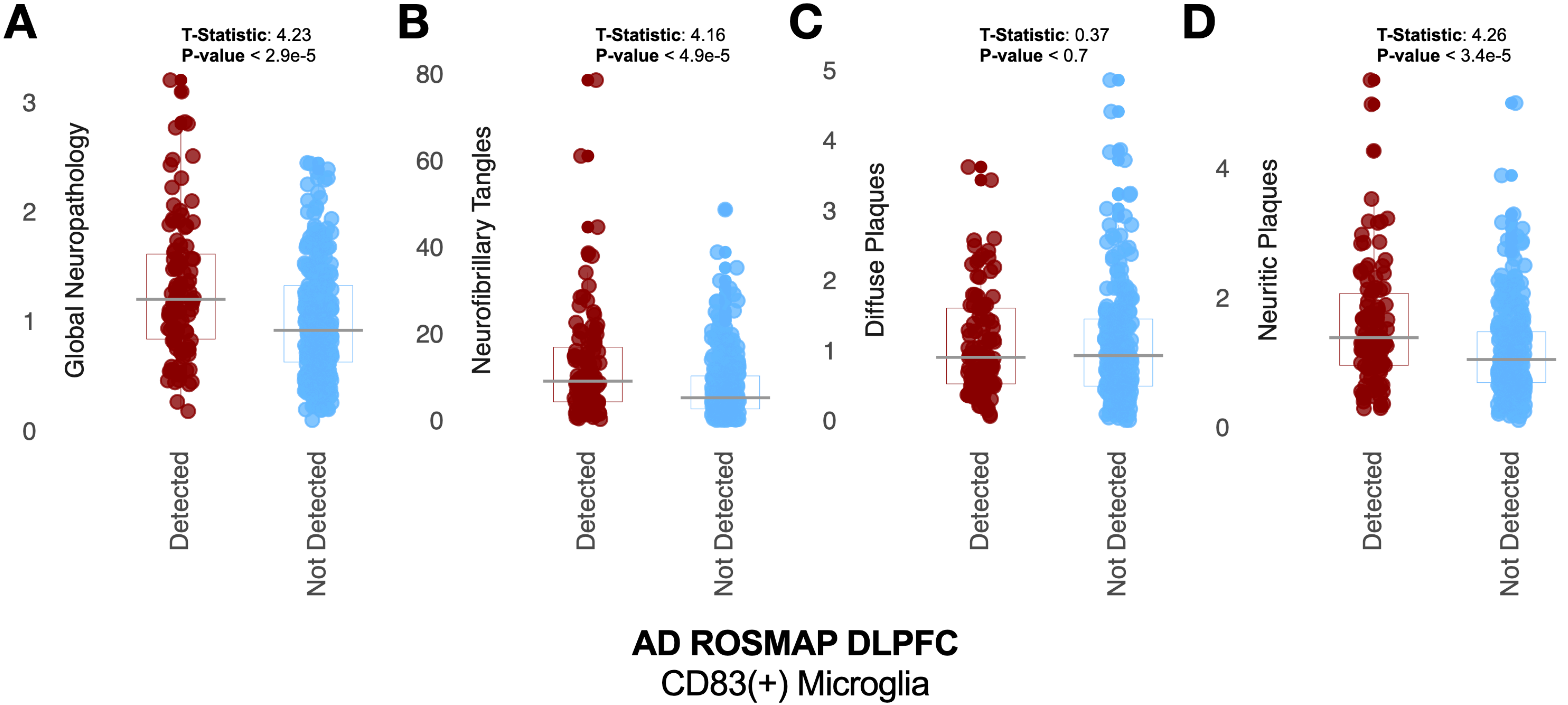

Supplementary Figure 5: Boxplot of neuropathological traits in the total unique set of AD subjects profiled in both Fujita-ROSMAP and Mathys-ROSMAP DLPFC studies, stratified by detection of differentially abundant CD83(+) microglia.

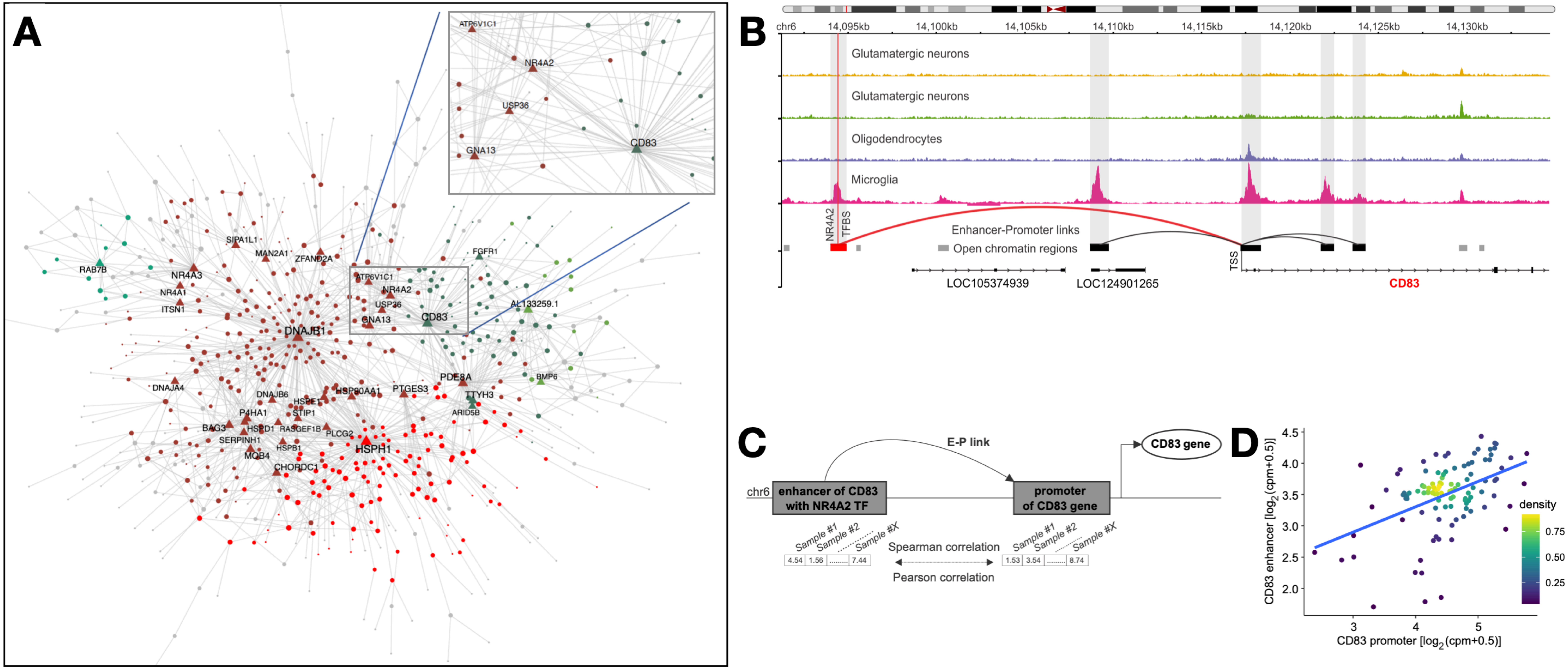

Supplementary Figure 6: Regulatory relationship between CD83 and NR4A2. **(A)** CD83(+) microglial networks demonstrate direct connectivity between CD83 and NR4A2. **(B)** Epigenomic landscape around CD83 gene body within major brain cell types. Normalized genomics tracks demonstrate the complex cell-specific regulation of CD83 via enhancer-promoter links. **(C)** Validation experiment investigating ABC-linked enhancer with presence of bound transcription factor NR4A2. **(D)** Correlation between chromatin accessibility at the CD83 promoter and predicted enhancer using chromatin accessibility data from 107 microglial ATAC-seq samples[41].
