# Supplementary material for: Single cell transcriptomes and multiscale networks from persons with and without Alzheimer’s disease
Source: Nat Commun. 2024 Jul 10;15:5815. doi: 10.1038/s41467-024-49790-0 (PMC11237088; doi:10.1038/s41467-024-49790-0)
Supplement: Supplementary file 3 — Description for Additional Supplementary Files [file 41467_2024_49790_MOESM3_ESM.pdf]

Description for Additional Supplementary Files

File Name: Supplementary Dataset 1
Description: Cell type annotation, cluster labels and DA population distribution. Diagnosis enrichment for each DA cluster.

File Name: Supplementary Dataset 2
Description: DEGs for each cell type by two different comparisons (DA vs non-DA; AD vs Controls). Genes are reported if it's differentially expressed in either comparison.

File Name: Supplementary Dataset 3
Description: eQTL summary statistics in each cell type.

File Name: Supplementary Dataset 4
Description: eQTL analyses for CR1 expression in oligodendrocytes and rs679515 association with hematocrit. chr1:207677194 / CR1 network and molecular functional enrichments of the network.

File Name: Supplementary Dataset 5
Description: Major allele dosage of rs679515 association with CR1 expression in oligodendrocytes and hematological traits in ROSMAP cohort.

File Name: Supplementary Dataset 6
Description: DA9 / CD83(+) microglia signature genes. Only those with absolute $\log_2$FC > 0.5 are reported.

File Name: Supplementary Dataset 7
Description: Gene set enrichment analysis of 308 DA9 / CD83(+) microglia hub genes

File Name: Supplementary Dataset 8
Description: DEGs for the DA vs non-DA microglial cells in ROSMAP snRNA-seq data. Only those with absolute $\log_2$FC > 0.5 are reported.

File Name: Supplementary Dataset 9
Description: Phenotypic associations with CD83(+) microglia

File Name: Supplementary Dataset 10
Description: Differential protein abundance between AD subjects with and without CD83(+) microglia (Transverse colon)
