# Supplementary material for: Single cell transcriptomes and multiscale networks from persons with and without Alzheimer’s disease
Source: Nat Commun. 2024 Jul 10;15:5815. doi: 10.1038/s41467-024-49790-0 (PMC11237088; doi:10.1038/s41467-024-49790-0)
Supplement: Supplementary file 14 — Reporting Summary [file 41467_2024_49790_MOESM14_ESM.pdf]

nature portfolio

Corresponding author(s): Benjamin Readhead

Last updated by author(s): 05/30/2024

Reporting Summary

Nature Portfolio wishes to improve the reproducibility of the work that we publish. This form provides structure for consistency and transparency in reporting. For further information on Nature Portfolio policies, see our Editorial Policies and the Editorial Policy Checklist.

Statistics

For all statistical analyses, confirm that the following items are present in the figure legend, table legend, main text, or Methods section.

n/a	Confirmed	
☐	☒	The exact sample size (*n*) for each experimental group/condition, given as a discrete number and unit of measurement
☐	☒	A statement on whether measurements were taken from distinct samples or whether the same sample was measured repeatedly
☐	☒	The statistical test(s) used AND whether they are one- or two-sided<br>*Only common tests should be described solely by name; describe more complex techniques in the Methods section.*
☐	☒	A description of all covariates tested
☐	☒	A description of any assumptions or corrections, such as tests of normality and adjustment for multiple comparisons
☐	☒	A full description of the statistical parameters including central tendency (e.g. means) or other basic estimates (e.g. regression coefficient) AND variation (e.g. standard deviation) or associated estimates of uncertainty (e.g. confidence intervals)
☐	☒	For null hypothesis testing, the test statistic (e.g. *F*, *t*, *r*) with confidence intervals, effect sizes, degrees of freedom and *P* value noted<br>*Give P values as exact values whenever suitable.*
☒	☐	For Bayesian analysis, information on the choice of priors and Markov chain Monte Carlo settings
☒	☐	For hierarchical and complex designs, identification of the appropriate level for tests and full reporting of outcomes
☐	☒	Estimates of effect sizes (e.g. Cohen's *d*, Pearson's *r*), indicating how they were calculated

Our web collection on statistics for biologists contains articles on many of the points above.

Software and code

Policy information about availability of computer code

Data collection	Analyses were performed using R software for statistical computing
Data analysis	Analyses were performed using R software for statistical computing

For manuscripts utilizing custom algorithms or software that are central to the research but not yet described in published literature, software must be made available to editors and reviewers. We strongly encourage code deposition in a community repository (e.g. GitHub). See the Nature Portfolio guidelines for submitting code & software for further information.

Data

Policy information about availability of data

All manuscripts must include a data availability statement. This statement should provide the following information, where applicable:

- Accession codes, unique identifiers, or web links for publicly available datasets
- A description of any restrictions on data availability
- For clinical datasets or third party data, please ensure that the statement adheres to our policy

Provide your data availability statement here.

Research involving human participants, their data, or biological material

Policy information about studies with human participants or human data. See also policy information about sex, gender (identity/presentation), and sexual orientation and race, ethnicity and racism.

Reporting on sex and gender	All analyses were performed on post-mortem, deidentified molecular data. This study was focused on identifyingmolecular networks associated with Alzheimer's disease. Sex was included as a covariate for most analyses and explicitly considered when examining whether CD83(+) microglia are sex-associated.
Reporting on race, ethnicity, or other socially relevant groupings	This study is based on post-mortem, deidentified molecular data generated from biosamples collected by several large clinicopathological studies of ageing and Alzheimer's disease. Participant's self-reported race was used as a covariate in relevant analyses, however these cohorts are predominantly comprised of caucasian individuals, so we did not perform any race-stratified analyses.
Population characteristics	We have outlined the subject characteristics of the three cohorts reported on in this study in Table 1 (Banner SFG cohort), Table 2 (Fujita-ROSMAP) and Table 3 (Mathys-ROSMAP)
Recruitment	Our study reports on data generated from deceased participants that were recruited into the Banner Sun Health Research Institute's Brain & Body Donation Program and the Religious Orders Study and Memory & Ageing Program. These subjects were not recruited by our study.
Ethics oversight	We did not require an IRB protocol as we were working with deidentified data from deceased individuals

Note that full information on the approval of the study protocol must also be provided in the manuscript.

Field-specific reporting

Please select the one below that is the best fit for your research. If you are not sure, read the appropriate sections before making your selection.

[x] Life sciences   [ ] Behavioural & social sciences   [ ] Ecological, evolutionary & environmental sciences

For a reference copy of the document with all sections, see nature.com/documents/nr-reporting-summary-flat.pdf

Life sciences study design

All studies must disclose on these points even when the disclosure is negative.

Sample size	We aimed to generate data from at least 100 subjects to power probabilistic causal network inference approaches that we planned to use
Data exclusions	N/A
Replication	We incorporated externally generated data from the ROSMAP-Fujita and ROSMAP-Mathys snRNAseq data sets which we used to verify whether our key findings were also evident in additional data sets
Randomization	N/A
Blinding	N?A

Reporting for specific materials, systems and methods

We require information from authors about some types of materials, experimental systems and methods used in many studies. Here, indicate whether each material, system or method listed is relevant to your study. If you are not sure if a list item applies to your research, read the appropriate section before selecting a response.

Materials & experimental systems

n/a	Involved in the study	
[x]	[ ]	Antibodies
[x]	[ ]	Eukaryotic cell lines
[x]	[ ]	Palaeontology and archaeology
[x]	[ ]	Animals and other organisms
[x]	[ ]	Clinical data
[x]	[ ]	Dual use research of concern
[x]	[ ]	Plants

Methods

n/a	Involved in the study	
[x]	[ ]	ChIP-seq
[x]	[ ]	Flow cytometry
[x]	[ ]	MRI-based neuroimaging

Plants

Seed stocks	N/A (Clicking on n/a for plants above results in these blank boxes appearing)
Novel plant genotypes	N/A
Authentication	N/A
